# Structural and Biochemical Characterization of a Cold-Active PMGL3 Esterase with Unusual Oligomeric Structure

**DOI:** 10.3390/biom11010057

**Published:** 2021-01-05

**Authors:** Konstantin M. Boyko, Mariya V. Kryukova, Lada E. Petrovskaya, Elena A. Kryukova, Alena Y. Nikolaeva, Dmitry A. Korzhenevsky, Galina Yu. Lomakina, Ksenia A. Novototskaya-Vlasova, Elizaveta M. Rivkina, Dmitry A. Dolgikh, Mikhail P. Kirpichnikov, Vladimir O. Popov

**Affiliations:** 1 Bach Institute of Biochemistry, Research Center of Biotechnology of the Russian Academy of Sciences, 119071 Moscow, Russia; vpopov@inbi.ras.ru; 2 Kurchatov Complex of NBICS-Technologies, National Research Centre “Kurchatov Institute”, 123182 Moscow, Russia; mar-1-ya@yandex.ru (M.V.K.); aishome@mail.ru (A.Y.N.); igra-voina@yandex.ru (D.A.K.); 3 Department of Bioengineering, Shemyakin & Ovchinnikov Institute of Bioorganic Chemistry, Russian Academy of Sciences, 117997 Moscow, Russia; kelen.kryukova@yandex.ru (E.A.K.); dolgikh@nmr.ru (D.A.D.); kirpichnikov@inbox.ru (M.P.K.); 4 Department of Chemistry, Lomonosov Moscow State University, 119991 Moscow, Russia; lomakinagalina@yahoo.com; 5 Department of Fundamental Sciences, Bauman Moscow State Technical University, 105005 Moscow, Russia; 6 Laboratory of Soil Cryology, Institute of Physicochemical and Biological Problems in Soil Science, Russian Academy of Sciences, 142290 Pushchino, Russia; nksusha@gmail.com (K.A.N.-V.); elizaveta.rivkina@gmail.com (E.M.R.); 7 Department of Biology, Lomonosov Moscow State University, 119234 Moscow, Russia

**Keywords:** PMGL3 esterase, HSL family, tetramer, dimer, GDSAG subfamily, mutagenesis, cold-active proteins

## Abstract

The gene coding for a novel cold-active esterase PMGL3 was previously obtained from a Siberian permafrost metagenomic DNA library and expressed in *Escherichia coli*. We elucidated the 3D structure of the enzyme which belongs to the hormone-sensitive lipase (HSL) family. Similar to other bacterial HSLs, PMGL3 shares a canonical α/β hydrolase fold and is presumably a dimer in solution but, in addition to the dimer, it forms a tetrameric structure in a crystal and upon prolonged incubation at 4 °C. Detailed analysis demonstrated that the crystal tetramer of PMGL3 has a unique architecture compared to other known tetramers of the bacterial HSLs. To study the role of the specific residues comprising the tetramerization interface of PMGL3, several mutant variants were constructed. Size exclusion chromatography (SEC) analysis of D7N, E47Q, and K67A mutants demonstrated that they still contained a portion of tetrameric form after heat treatment, although its amount was significantly lower in D7N and K67A compared to the wild type. Moreover, the D7N and K67A mutants demonstrated a 40 and 60% increase in the half-life at 40 °C in comparison with the wild type protein. *K*_m_ values of these mutants were similar to that of the wt PMGL3. However, the catalytic constants of the E47Q and K67A mutants were reduced by ~40%.

## 1. Introduction

Lipolytic enzymes which catalyze cleavage of the ester bonds in triacylglycerols are widely used in biotechnology including the food industry, fine chemical synthesis, production of detergents, and biofuel [1,2]. Lipases and esterases from extremophilic microorganisms (thermophilic, psychrophilic, halophilic, etc.) frequently possess unique structural and functional characteristics which enable them to operate in harsh environmental conditions [3]. These properties provide a foundation for the ongoing search for new representatives of the family and numerous studies devoted to their characterization.

In particular, many cold-active lipolytic enzymes demonstrate high catalytic efficiency and activity at low temperatures [4,5]. They can also catalyze reactions in the presence of high salt concentration and organic solvents. However, low thermal stability usually limits their broad industrial application.

Similar to other lipolytic enzymes, cold-active lipases and esterases possess common α/β hydrolase fold which consists of a catalytic and a cap domain [2,6]. The former includes a β-sheet of 5–11 β-strands surrounded by α-helices and contains a catalytic triad of Ser-His-Asp/Glu residues. In comparison with more thermostable analogues, the molecules of cold-active enzymes typically demonstrate smaller hydrophobic core, lower number of stabilizing interactions, the presence of long extended loops and greater number of exposed hydrophobic residues at the protein surface [7,8].

Being a habitat for diverse cold-adapted microorganisms, Siberian permafrost is a unique environment which is characterized by the presence of permanently frozen ground, limited accessibility of organic matter, low water activity and other factors [9,10,11]. Previously, we have described several lipolytic enzymes from the permafrost bacterium *Psychrobacter cryohalolentis* K5^T^ [12,13,14]. Furthermore, we performed screening of the metagenomic DNA library obtained from the permafrost-derived microcosm that resulted in production and characterization of new esterases PMGL2 and PMGL3 belonging to the bacterial hormone sensitive lipase (bHSL, EC 3.1.1.79) family [15,16]. The PMGL3 esterase demonstrated low thermal stability with rapid inactivation upon incubation at 40 °C, which is typical for the cold-active enzymes. In particular, the enzyme was shown to possess a dimeric state in solution, with a tendency to oligomerize upon heating [17].

Here, we comprehensively studied the biochemical properties of the wild type PMGL3 and determined its 3D structure. Surprisingly, it has a tetrameric architecture in a crystal, with a subunit’s fold common to the members of the GDSAG motif subfamily of the bHSL family in spite of the low amino acid similarity with structurally characterized esterases. Moreover, the crystal tetramer of PMGL3 has a unique architecture compared to other known tetramers of bHSLs. In order to study an effect of tetramerization on biochemical properties of the enzyme and to identify structural motifs important for tetramerization, we carefully analyzed dimeric and tetrameric interfaces and performed site-directed mutagenesis of the residues possibly stabilizing tetrameric contacts through formation of salt bridges. Our results demonstrated that mutations of non-conserved D7 and K67 residues mostly affected the tetramerization and the thermal stability of PMGL3.

## 2. Materials and Methods

### 2.1. Protein Purification, Crystallization and Data Collection

DNA manipulations were performed by standard techniques using enzymes from Thermo Fisher Scientific. Expression and purification of the wild type PMGL3 was described previously [16,17]. The mutant genes were constructed by two-step Splicing by Overlap Extension (SOE)-PCR using *Pfu* DNA polymerase and primers listed in Table S1. The resulting products were cloned into the pET32a vector and mutations were confirmed by DNA sequencing (Evrogen). The mutant proteins were obtained as described for the wild type PMGL3 [17].

The initial crystallization screening of the wild type PMGL3 was performed with a robotic crystallization system (Rigaku, USA) and commercially available 96-well crystallization screens (Hampton Research, USA) at 15 °C using the sitting drop vapor diffusion method. The protein concentration was 10 mg/mL in 100 mM Tris, pH 7.8, NaCl 50 mM. The optimization of the initial crystallization conditions was done using the hanging drop vapor diffusion method. The optimized crystallization solution contained 0.1 M sodium citrate pH 5.6, 20% isopropanol, 20% PEG 4000.

Immediately before data collection, the crystal of PMGL3 was briefly soaked in the mother liquor containing 25% glycerol as a cryoprotectant. The crystal was then flash-cooled to 100 K in liquid nitrogen. The X-ray diffraction data were collected at the ID30A-3 beamline [18] of ESRF synchrotron (Grenoble, France). Due to the rod-shaped form and small size of the crystal, data were collected in a helical mode. Data collection strategy was calculated with BEST [19]. The data were indexed, integrated and scaled using XDS package [20]. Based on the L-test [21], the dataset was not twinned and the program Pointless [22] suggested the C2 space group.

The data collection and processing statistics are summarized in Table 1.

### 2.2. Structure Solution and Refinement

The structure of the wild type PMGL3 was solved by the molecular replacement method using the MOLREP program [23] with the atomic coordinates of the HSL-like esterase EstE5 from a metagenomic library (PDB ID 3FAK) as a starting model. The refinement of the structure was carried out using the REFMAC5 program of the CCP4 suite [24]. The resolution was cut to 2.30 Å during the refinement due to a better electron density and R-factors. TLS and NCS were introduced during the refinement. The visual inspection of electron density maps and the manual rebuilding of the model were carried out using the COOT interactive graphics program [25]. In the final model, an asymmetric unit contained two independent copies (subunits A and B) of the protein each consist of 302 residues together with total 197 solvent molecules. The last 10 C-terminal residues, including hexahistidine tag (His-tag), were invisible in electron density of both subunits possibly due to their high flexibility. The superposition of two protein molecules from the asymmetric unit gave an RMSD between Cα atoms of less than 0.2 Å, indicating high similarity between the molecules.

### 2.3. Structure Analysis and Validation

The visual inspection of the structures was carried out using the COOT program [25] and the PyMOL Molecular Graphics System, Version 1.9.0.0 (Schrödinger, USA). Amino acid sequence alignment was made with BLAST [26] and Clustal Omega [27]. The structure comparison and superposition were made using the PDBeFold program [28], while contacts were analyzed using the PDBePISA [29] and WHAT IF software [30]. LigPlot [31] was used to analyze molecular interfaces.

### 2.4. Biochemical Characterization

Esterase activity assay was conducted at 30 °C as described previously [17] using 0.25 mM *p*-nitrophenyl butyrate (*p*-NPB) as a substrate in 50 mM Tris-HCl pH 8.0, 100 mM NaCl. Kinetic parameters of the reaction were determined from Michaelis-Menten equation using *p*-NPB substrate concentrations ranging from 0.2 to 2 mM. The values for *K_m_* and *V_max_* were estimated by nonlinear regression using Origin 8 software. The thermal stability of the enzymes (2 μg/mL) was determined by measuring residual activity under standard conditions after incubation at 40 °C for 0–28 min. The half-time of inactivation, *t*_1/2_, was calculated from time course study as first-order kinetics.

### 2.5. Circular Dichroism Spectra

CD spectra were recorded on a Chirascan CD spectrometer (Applied Photophysics, UK) at 188–320 nm at a constant time of 3 s, scan speed 10 nm/min. Spectra were measured at 20 °C in the protein samples with concentration 0.6 mg/mL in 20 mM K_2_PO_4_, 150 mM KF. The content of the secondary structure elements was calculated with DichroWeb service using the CDSSTR method and reference data sets 4, 7, SP175, SMP180.

### 2.6. Size Exclusion Chromatography

Size exclusion chromatography (SEC) of the purified proteins was conducted on a Superdex 200 10/300 GL column (GE Healthcare) at a flow rate of 0.4 mL/min in 100 mM Tris-HCl, pH 8.0, 150 mM NaCl.

## 3. Results

### 3.1. Biochemical Characterization of PMGL3

In our previous work, we, particularly, performed expression and brief characterization of PMGL3 catalytic properties which demonstrated that it is an esterase with temperature optimum at 30 °C and low thermal stability [16]. The comprehensive analysis of the esterase activity of PMGL3 is provided in the current paper.

We have shown that PMGL3 was highly active up to 0.25 M NaCl while higher salt concentrations (0.5–1.75 M) partially inhibited the enzyme (Table 2). It was drastically inhibited by Zn^2+^, Ni^2+^, Cu^2+^ and Co^2+^, whereas addition of Ca^2+^, Mg^2+^ and Mn^2+^ did not produce significant impact on the catalytic activity of PMGL3 (Table 3). Incubation in the presence of 1 mM PMSF decreased the activity of PMGL3 to 13% of the initial level, while 5 mM PMSF completely inhibited it. The strong inhibitory effect confirmed that PMGL3 belongs to the serine hydrolase family. In the presence of 1 mM EDTA the activity of the enzyme was not affected.

PMGL3 demonstrated relatively good tolerance of nonionic detergents Triton X-100 and Tween 20, while in the presence of zwitterionic surfactant CHAPS its activity slightly increased (Table 4). The harsh anionic detergent SDS inactivated the enzyme almost completely. The esterase activity of PMGL3 was moderately inhibited in the presence of methanol and ethanol and strongly decreased by acetonitrile and dimethylformamide (Table 5). Addition of 5% dimethylsulfoxide was well tolerated. In accordance with the absence of disulfide bonds in the protein [17], activity of PMGL3 was not affected by the presence of β-mercaptoethanol or DTT in the reaction mixture.

### 3.2. Tetramerization of PMGL3 and Its Relation to Enzyme Stability

**Figure 1:**
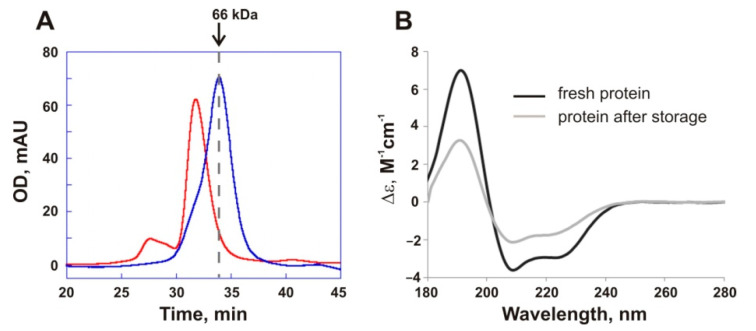
(**A**) Size exclusion chromatography (SEC) analysis of the freshly isolated sample of PMGL3 (blue line) and the sample after storage for 3 months at 4 °C (red line) on a Sephadex G-200 column calibrated by carbonic anhydrase (29 kDa), ovalbumin (43 kDa), bovine serum albumin (66 kDa) and thyroglobulin (669 kDa). (**B**) Circular dichroism (CD) spectra of the freshly isolated PMGL3 (black line) and the protein after storage for 3 months at 4 °C (light grey line). The spectra were measured at 20 °C at a constant time of 3 s, scan speed 10 nm/min and step of 1 nm.

Previously, we demonstrated that PMGL3 possesses low thermal stability. After incubation for 60 min at 40 °C PMGL3 lost ~73% of its lipolytic activity and treatment at 50 °C led to its complete inactivation [17]. Furthermore, we have noticed that prolonged (for several weeks) storage of the enzyme at 4 °C resulted in a dramatic loss of its activity. For example, after 1 month storage at 4 °C PMGL3 demonstrates 50% of catalytic activity in comparison with freshly isolated protein, and after 3 months it loses 90% of activity. Electrophoretic analysis and concentration measurements after centrifugation demonstrated that the protein was not subjected to proteolytic digestion or precipitation (data not shown). To elucidate the structural basis of this phenomenon, we performed gel filtration analysis of the freshly isolated sample of the enzyme and the sample after storage for 3 months at 4 °C (Figure 1A). Clearly, the fresh protein sample is represented mainly by the dimeric form (elution time ~34 min). The position of the main peak in the sample after storage presumably corresponds to the tetramer (elution time ~32 min). Higher molecular weight aggregates were also present in the sample after storage.

Secondary structure content of the protein samples was estimated by CD spectroscopy. These studies revealed large spectral changes of the PMGL3 sample after storage in comparison with the freshly isolated protein that can be explained by partial unfolding of the molecule (Figure 1B, Table S1). The number of alpha-helices in the protein sample after storage decreased by 20%, from 37 to 17, and the content of beta-sheets increased correspondingly (from 19 to 32%). A small increase in irregular structure content (by ~6%) was also observed. Therefore, we have concluded that inactivation of PMGL3 in the course of prolonged storage at 4 °C is accompanied with the loss of the native secondary structure and subsequent transition of the protein from the dimeric to tetrameric and higher oligomeric aggregates. The presence of tetramers and aggregates was observed previously in the samples of PMGL3 which were incubated for 1 h at 40 °C [17].

### 3.3. PMGL3 Forms Two Types of Tetramers in a Crystal

**Figure 2:**
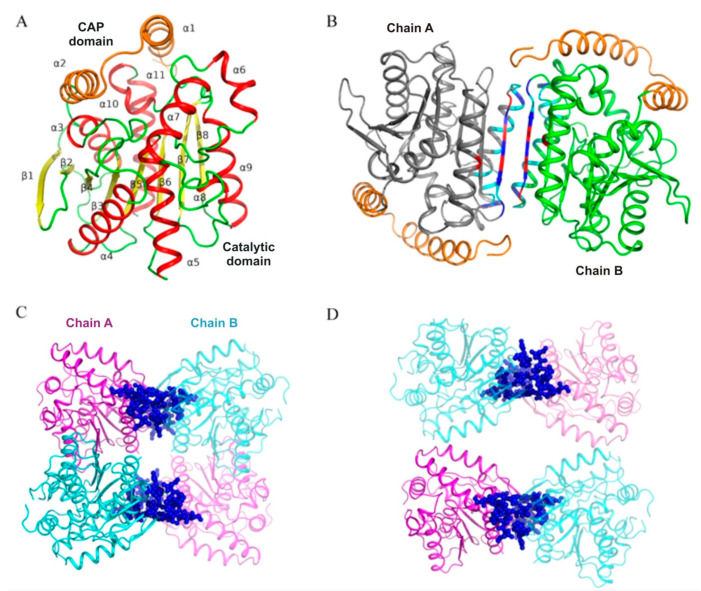
Structure of PMGL3 esterase. (**A**) Cartoon diagram of the PMGL3 subunit. Secondary structure elements are color coded (α-helix—red; β-strand—yellow; loop—green). CAP-domain is highlighted in orange. (**B**) PMGL3 dimer from the asymmetric unit. Subunits are colored by chain. CAP-domain is highlighted in orange. Residues forming the dimeric interface are color coded (blue–hydrogen bonds, red–salt bridges, orange–hydrophobic interactions). Crystallographic tetramers—T1 (**C**) and T2 (**D**) of PMGL3. Tetramers are colored by chains–chain A from the asymmetric unit is in magenta, while chain B is in cyan. Symmetry mates of the chains from the asymmetric unit have the same color and are semi-transparent for clarity. Residues of the interdimeric interface are shown as blue spheres.

To shed light on a structural basis of different oligomeric states of PMGL3, we successfully crystallized the protein and solved its 3D structure. PMGL3 subunit has a typical α/β bacterial HSL-like fold, consisting of two canonical domains—a CAP-domain and a catalytic domain [32] (Figure 2A). The CAP domain (residues 1–42) contains two α-helices (α1 and α2), while the large catalytic domain contains 8 β-strands (parallel β1 and β3-β8 as well as antiparallel to them β2) surrounded by 9 alpha-helices (α3-α11). The secondary structure content of PMGL3 in a crystal (47% α-helices and 14% β-strands) is more similar to the structure of a native protein in solution (37% α-helices and 19% β-strands based on CD spectra, Table S1), rather than to a partially unfolded one (17% and 32%, accordingly).

**Figure 3:**
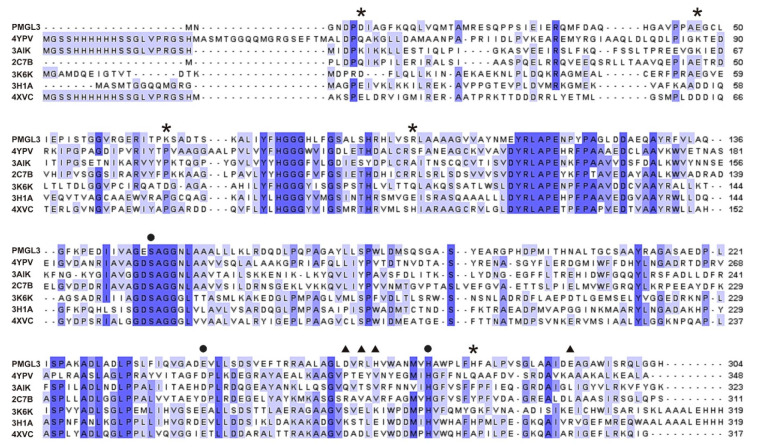
Multiple sequence alignment of PMGL3 and homologous esterases with known three-dimensional structure from the HSL family produced with Clustal Omega [27]. Hereinafter, corresponding PDB IDs are shown instead of enzyme names for clarity. The extent of amino acid sequence conservation is depicted in the grades of blue. Black circles mark amino acid residues comprising the catalytic triad. Black triangles and asterisks indicate the positions of charged residues which belong to the dimeric and interdimeric interfaces correspondingly. The PDB codes replacing enzymes names are defined in Table 7 below.

Two PMGL3 subunits within an asymmetric unit form a homodimer, which is consistent with the current and previous SEC data [17]. The area that becomes buried upon PMGL3 dimer formation is 930 Å^2^, which comprises about 8% of the total surface area of each subunit. The dimeric interface is stabilized by 10 hydrogen bonds, 4 salt bridges as well as by hydrophobic interactions (Table S3, Figure S3), thus involving α6, α9, α11 and β8 from each subunit and not affecting their CAP-domains (Figure 2B and Figure 3).

Further contact analysis revealed two possible tetrameric forms (T1 and T2) of PMGL3 within a crystal (Figure 2C,D). T1 tetramer can be regarded as a dimer of dimers from the asymmetric unit and, thus, has two types of intermolecular interfaces–the dimeric interface, described above, and interdimeric interface (Figure 2C). The latter has about 6% of buried surface per each subunit and is fastened by 8 hydrogen bonds (residues M1, N2, K67, A69, T71), 10 salt bridges (residues D7, E47, K67, R97, H279) as well as by hydrophobic interaction (residues G48, T65, P66, D70, Q283, V284, L287). This interface involves the CAP-domain (helices α1 and α3), α10 and α11 of each PMGL3 subunit. T2 is based on only one protein chain from the asymmetric unit, however it has the same interdimeric interface (Figure 2D) due to symmetry operations resulting in the formation of T2.

Analysis of tetramerization interfaces demonstrated that T1 has significantly greater buried area, number of polar interactions and free energy of assembly dissociation than T2 (Table 6). This indicates that T1 form is more stable and presumably the one found in solution, while T2 form is a result of crystal packing. Thus, we further focused on a study of T1 form (referred as tetramer hereinafter).

### 3.4. PMGL3 Active Site

Amino acid sequences of bacterial lipolytic enzymes belonging to the HSL family are characterized by the presence of several conserved motifs [33,34] being a part of the active site or located nearby. The conserved motif GxSxG which is characteristic for the bHSL family [33] (^147^GESAG^151^ in PMGL3) contains an invariant catalytic serine residue (Figure 3). Two remaining residues of the catalytic triad, E243 and H273, are located in PMGL3 in the conserved motifs ^240^GADE^243^ and ^296^HAW^305^. Noteworthy, PMGL3 contains a glutamic acid residue E243 instead of more common aspartate in the corresponding position. However, the substitution of the catalytic aspartate with a glutamic acid residue with longer side chain is not unique for bHSLs (e.g., esterase EstE5, PDB ID 3H1A; Figure 3). A characteristic motif HGGG, which is involved in hydrogen bonding interactions stabilizing the oxyanion hole [33], is conserved in PMGL3 as ^79^HGGG^82^. Finally, a conserved motif ^111^YRLA^114^ is also found in PMGL3.

**Figure 4:**
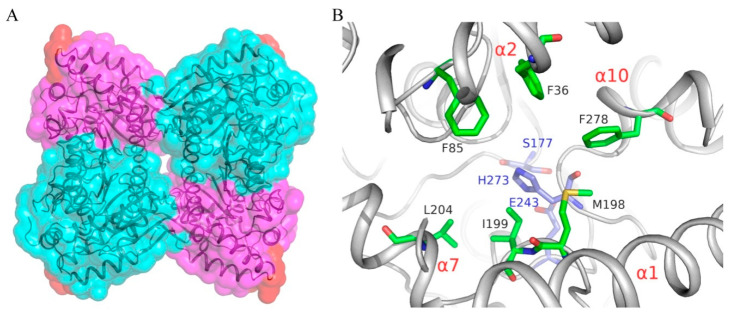
PMGL3 active site. (**A**) Four active sites of the PMGL3 tetramer facing the solution. Tetramer orientation and coloring is the same as in Figure 2C. Part of the CAP-domain forming “bag handle”-like arc above the active site entrance is colored in red. (**B**) Residues forming a neutral charge of the active site entrance (green) and the catalytic triad (blue). Corresponding α-helices are labeled in red. View from the entry to the active site.

PMGL3 tetramer has four active sites each made by the residues which belong to one subunit and are buried into the protein molecule. Entrances to the active site are oriented outwards the tetramer and accessible to solution (Figure 4A). The oval entrance of about 13 × 7 Å is partly covered from the protein surface by the CAP-domain, allowing, however, access for the substrate through two large holes formed by a “bag handle”-like arc being composed of α1, α2 and the loop connecting these helices. The entrance has nearly neutral charge due to the sidechains of residues F36, F85, M198, I199, L204 and F278 (Figure 4B). The arc has, however, a positive patch made by R22, which could facilitate proper substrate orientation. Catalytic residues of PMGL3 are connected to each other via hydrogen bond network, which includes S177-E243 and E243-H273 interactions.

### 3.5. Comparison of PMGL3 Structure with the Structures of Homologous Enzymes

**Figure 5:**
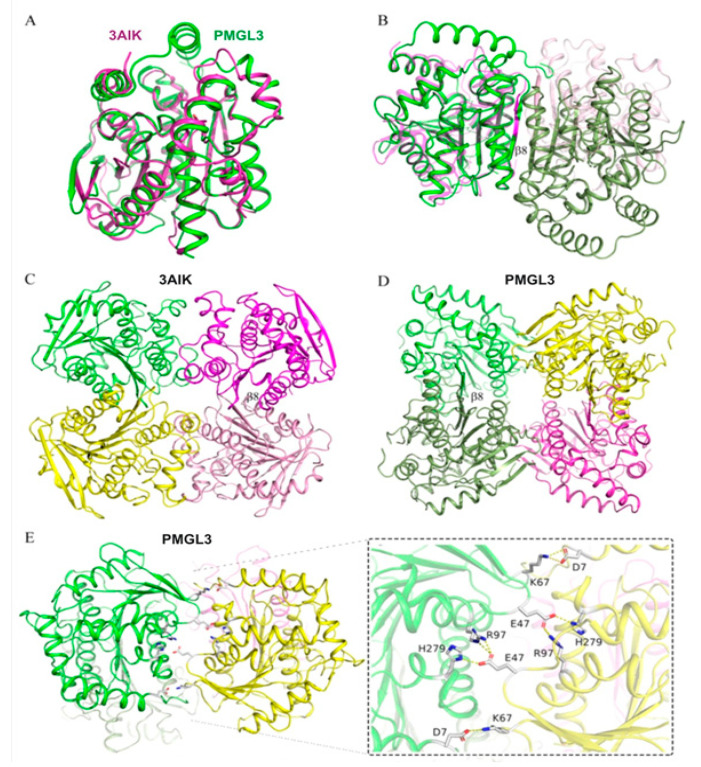
Comparison of PMGL3 structure with the structures of homologous enzymes. (**A**) Superposition of PMGL3 subunit (green) with that of 3AIK (magenta). Orientation is similar to Figure 2A. (**B**) Superposition of PMGL3 (subunits are colored in shades of green) and 3AIK (subunits are semitransparent and colored in shades of magenta) homodimers. Superposition was made by one subunit of the dimer. Two adjacent β8 strands are labelled. Comparison of 3AIK (**C**) and PMGL3 (**D**) tetramers colored by chain. Corresponding dimers have the same color as on the panel B and adjacent β8 strands are labelled. (**E**) Position of PMGL3 residues within the interdimeric contact which were mutated in this study (view from the top of the panel D). Corresponding salt bridges are indicated in a zoomed view. A cartoon color scheme is the same as on the panel D.

The bacterial HSL family is divided into two subfamilies based on G(D/E)SAG or G(T/X)SAG motifs of the active site, with various amino acids present in the second position of the latter group [34,35,36]. As PMGL3 contains ^147^GESAG^151^ motif, comparison of its structure with those of homologue enzymes revealed a number of structurally similar bHSL enzymes from GDSAG subfamily (Table 7). Structure superposition demonstrated that secondary structure elements of PMGL3 subunit have similar spatial positions to those of homologous enzymes (Figure 5A), in spite of their amino acid identity comprising less than 40% (Table 7). The differences were found only within the looped regions. The active site residues of PMGL3 also possess a canonical conformation.

Oligomeric quaternary structure is a characteristic feature of many esterases belonging to the bHSL family [35,36,37,38] with dimeric state being quite common. It was proposed previously that dimerization interface of bHSLs differs between enzymes of GDSAG and GTSAG subfamilies [35,36]. Thus, for GDSAG subfamily the dimeric interface is mainly formed by the interactions between two antiparallel β8-strands from each of the subunits and doesn’t involve the CAP-domain [39,40,41]. This is, indeed, true for the PMGL3 dimer (Figure 2B).

A diversity of oligomeric states for different bHSLs is demonstrated in Table 7, where 3H1A, 4XVC, and 4YPV are monomers, 2C7B and 3AIK–tetramers, 4C88–might have different oligomeric states, while the others are dimers. Based on a pairwise structural comparison of dimeric states, the enzymes from Table 7 can be clustered into a number of groups. Thus, PMGL3 and 3K6K fall into group 1 as having similar spatial architecture with RMSD of 2.6 Å, while dimers of 2C7B and 3AIK (from corresponding tetramers) as well as 5HC3, 4WY5, 4WY8 and 4J7A fall into another group 2, being similar to each other, but differ from PMGL3 dimer significantly (Figure 5B). Finally, 4C88 and 6AAE dimers seem to have a unique architecture. Despite the totally different dimeric organization between groups, enzymes from group 2 resemble to a certain extent the architecture of PMGL3 dimer (in both groups dimers are formed via interaction of β8 strands of each subunit); however, in group 2 the subunits are mutually shifted along the axis parallel to β8 strand of one subunit and rotated about 40° around the axis perpendicular to this β8 strand. Consequently, a comparison of PMGL3 tetramer with those of 3AIK and 2C7B (group 2) revealed an absolutely different spatial arrangement of subunits (Figure 5C,D), whereas 3AIK and 2C7B tetramers are quite alike. Moreover, Protein Data Bank analysis revealed a number of other tetrameric bHSLs similar to 3AIK (e.g., PDB ID 3ZWQ, 1JKM, etc), but failed at all to find tetramers similar to that of PMGL3. Interestingly, even if the dimerization interface involves β8 strands of each subunit (Figure 5C,D), differences in this interface lead to completely different tetrameric structures. Thus, we can assume that the crystallographic PMGL3 tetramer has a unique spatial architecture.

### 3.6. Site-Directed Mutagenesis of PMGL3 Residues Presumably Participating in Tetramer Formation

With the aim of studying the effect of PMGL3 tetramerization on its thermal stability, we mutated five charged residues D7, E47, K67, R97, and H279, which participate in the formation of all the salt bridges in tetrameric interface of the protein. The corresponding mutants which contained substitutions of these residues for the uncharged ones (D7N, E47Q, K67A, R97Q and H279A) were constructed by site-directed mutagenesis and produced in *E. coli* cells in the soluble form, similarly to the wild type protein. Preliminary biochemical experiments demonstrated that mutants R97Q and H279A did not possess any detectable activity; therefore, subsequent studies were performed with the three other mutants.

**Figure 6:**
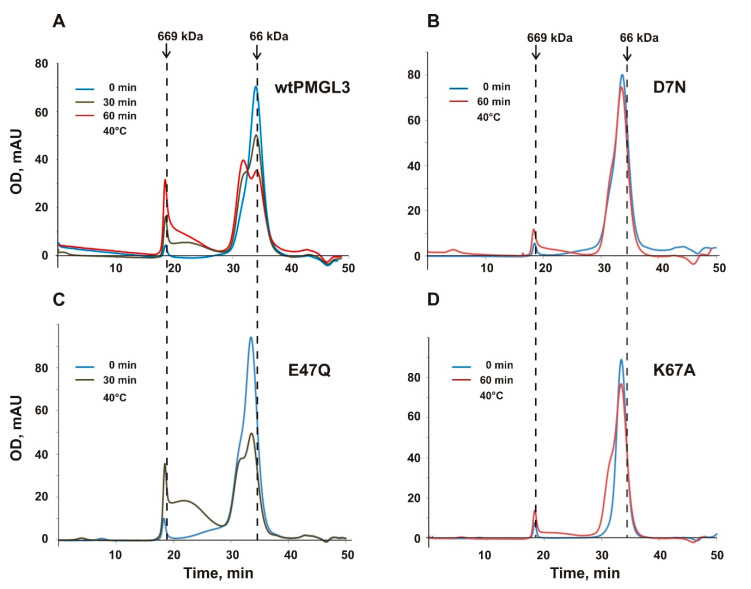
SEC analysis of wtPMGL3 (**A**) and the mutant proteins (**B**–**D**) (fresh samples and after incubation for 30 min and/or 1 h at 40 °C) on Sephadex G-200 column calibrated by carbonic anhydrase (29 kDa), ovalbumin (43 kDa), BSA (66 kDa) and thyroglobulin (669 kDa). Dashed lines indicate elution times of bovine serum albumin and thyroglobulin.

Analysis of CD spectra of the PMGL3 mutant variants demonstrated that their secondary structure exhibited small variation in comparison with that of the wild type PMGL3 (Figure S1 and Table S2). Chromatographic mobility of the mutants during the SEC analysis was also similar to the wild-type protein (Figure 6). Mobility of the main peak corresponded to the dimeric form while all samples (except K67A) contained small amount of the tetramer and the aggregated material. To access the effect of temperature on the monodispersity of the mutants, we repeated this analysis after their incubation at 40 °C. Upon this treatment, all mutant proteins still contained a portion of tetrameric form though in the D7N and K67A proteins it was lower in comparison with the wtPMGL3 (Figure 6 A,B,D). After incubation for 30 min at 40 °C, the E47Q mutant demonstrated the same amount of tetramer as wtPMGL3 while the amount of aggregated material was even greater (Figure 6C).

To compare the effect of temperature on the esterase activity of the mutants with that of the wild type PMGL3, we have measured their half-life times upon incubation at 40 °C (Figure S2 and Table 8). Mutants D7N and K67A demonstrated increased stability in comparison with the wild type protein (*t*_1/2_ 51 and 59 min correspondingly that is 1.4 and 1.6-fold higher than that of the wt PMGL3) while the E47Q mutant showed reduced stability (*t*_1/2_ 25.6 min).

The kinetic parameters for the wild type PMGL3 and its mutant variants were studied with *p*-NPB as a substrate at 30 °C (Table 8). Obtained data demonstrate that the *K_m_* values of the mutants were close to the value for the wild type PMGL3. In contrast to that, *V_max_* and the catalytic constant *k_cat_* were reduced in E47Q and K67A mutants. Correspondingly, the catalytic efficiency of the mutant enzymes decreased by ~40% in comparison with wtPMGL3 while it was almost unchanged for D7N.

## 4. Discussion

**Figure 7:**
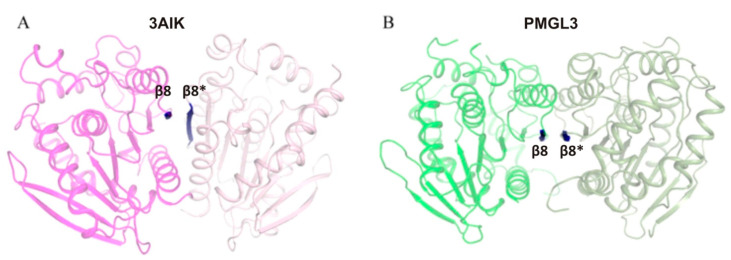
Comparison of dimeric interfaces in two proposed subclasses of GDSAG subfamily. (**A**) The dimeric interface of 3AIK. Dimer is colored by chains and oriented so as one β8 strand is almost perpendicular to the plane of image, β8-β8* interface is in blue, highlighted and labeled. (**B**) The dimeric interface of PMGL3. Color scheme and orientation procedure are similar to panel A.

In this work, we performed structural and biochemical characterization of the PMGL3 esterase, a novel member of the GDSAG motif subfamily of the bHSL family. The protein was obtained from the metagenomic DNA library constructed from the permafrost-derived microcosm. The PMGL3 demonstrated a typical fold for the bHSL family with however a unique tetrameric architecture. In many bHSLs, a β8 strand of the adjacent subunits is an important determinant of the dimeric interface. A major difference between GDSAG and GT/XSAG bHSL subfamilies is the mutual orientation of β8 strands within the dimeric interface. They are antiparallel in case of GDSAG subfamily [39,40,41] and about to perpendicular for GT/XSAG [35,36]. According to our results, we speculate that bHSLs of GDSAG subfamily with dimeric and/or tetrameric organization could be further subdivided based on details of their dimeric interface. In the structures of most members of this subfamily (e.g., 3ZWQ, 1JKM, 3AIK, 2C7B, etc.) the β8-β8* strands are not strictly antiparallel, but intercrosses at angle of about 40° (Figure 7A). In other cases (PMGL3, 3K6K), these strands are antiparallel and lie almost in the same plane (Figure 7B). This difference leads to completely different dimeric or tetrameric (if any) architectures. Noteworthy, structures of some bHSLs revealed that oligomeric forms could be organized completely without involvement of β8 strands (e.g., 4C88 and 6AAE). Further widening of structural “universe” of bHSLs could shed light on this issue.

Previously, we have shown that PMGL3 is a cold-active esterase with low thermal stability [16,17]. It exhibited increased potential for oligomerization and dramatic loss of activity upon incubation even at 40 °C. In the current work, we demonstrated that prolonged storage of PMGL3 at 4 °C also leads to enzyme inactivation which is accompanied with the loss of the native secondary structure content and formation of oligomers. Limited thermal stability is a characteristic feature of the cold-active enzymes [7,8]. Several strategies have been proposed to increase their half-life based on the directed evolution or rational engineering approaches [49,50]. The extension of knowledge about 3D structures of cold-active enzymes can promote such studies due to preliminary identification of the sites for potential modifications.

In our earlier work, two cysteine residues of PMGL3, C49 and C207, were mutated in order to improve enzyme properties. The former mutation led to accelerated thermoinactivation of the enzyme, and the latter one increased thermal stability of the mutant protein and reduced its tendency to oligomerization [17]. In the current paper, elucidation of the PMGL3 3D structure enabled us to explain this effect from a structural point of view. A slight decrease in temperature stability for C49V mutant could be a result of poor packing of the hydrophobic valine side chain in surrounding residues. Despite V49 can be modelled in this position without steric clashes, we could speculate that its placement is energetically less favorable than of the cysteine residue. In case of C207F substitution, the reason of improved thermostability seems to be clearer as large phenolic side chain of F207 might strengthen a hydrophobic core nearby, containing, in particular, residues L84, F85, W179 and L204.

The availability of the 3D structure also allowed us to identify residues participating in the formation of tetrameric interface, which accompanies thermal unfolding and inactivation of the enzyme. We have focused on mutagenesis of the charged residues, which stabilize the tetrameric interface via the salt bridges, which are the strongest among polar interactions. However, no one of the studied single mutations was sufficient to completely prevent tetramerization of PMGL3 upon heating, pointing to the effect of other (e.g., hydrophobic) stabilizing interactions that lead to the high stability of the tetramer. Significant decrease of the tetramer formation after incubation for 60 min at 40 °C was observed for D7N and K67A mutants while for E47Q a substantial amount of tetramer formed even after 30 min heating. In accordance with that, the inactivation half-time of the E47Q mutant was by 28% lower than that of the wild type PMGL3 while the half-lives of the D7N and K67A mutants exceeded it by 40 and 60% correspondingly.

Single mutations D7N, E47Q, and K67A did not significantly changed substrate affinity; however, *k_cat_* of E47Q and K67A was reduced. It should be mentioned that two of the mutations, R97Q and H279A led to inactivation of the enzyme. Based on structural analysis, both R97 and H279 are disposed close to each other and participate in a network of hydrogen bonds, which, presumably, supports the native conformation of the enzyme.

Formation of oligomers is a common feature of thermophilic and hyperthermophilic enzymes, which is known to contribute to their high thermal stability [51]. Thus, thermophilic esterases PestE from *Pyrobaculum calidifontis* VA1 [39] and EstE1 from metagenomic library [40] are tetramers in a crystal. Moreover, strengthening of oligomeric interfaces, in particular, by introduction of the salt bridges is considered an efficient bioengineering approach to enzyme stabilization [52,53]. This approach was successfully used to increase thermal stability of malate dehydrogenase [54], PyrR [55], αE7 carboxylesterase [56] and other proteins. However, if the partially unfolded proteins form oligomers then electrostatic interactions can lead to stabilization of aggregates and enzyme inactivation [57]. Based on the presence of interfaces to form tetramers in PMGL3 molecule, we supposed that oligomers of partially unfolded enzyme can be stabilized by these interfaces and demonstrated that mutations that disrupt tetrameric interactions can increase thermal stability of the enzyme. The obtained results can be used further to increase the thermal stability of PMGL3 and other esterases for their biotechnological application.

## 5. Conclusions

In summary, we provided detailed characterization of the catalytic properties of the cold-active esterase PMGL3 and elucidated its spatial structure. PMGL3 forms tetramers in a crystal and in solution upon prolonged storage or incubation at elevated temperatures. We demonstrated that in spite of overall high similarity of the subunit’s architecture to other members of GDSAG subfamily of bHSLs, PMGL3 possesses a unique tetramerization interface, which is a result of two possible means of dimerization in GDSAG subfamily. By altering charged residues, which strengthen this interface, we have shown their significance for tetramer formation and enzyme stability. Our study expands structural data and provides insights on possible stabilization strategies of the enzymes belonging to the bacterial HSL family.

## Figures and Tables

**Table 1:** Data collection, processing and refinement.

Diffraction Source	ID30A-3 (ESRF)
Wavelength (Å)	0.97
Temperature (K)	100
Detector	EIGER
Crystal-to-detector distance (mm)	117.55
Rotation range per image (°)	0.15
Total rotation range (°)	120
Space group	C2
*a*, *b*, *c* (Å)	103.80; 98.40; 75.20
α, β, γ (°)	90.00; 128.10; 90.00
Average mosaicity (°)	0.52
Resolution range (Å)	37.28–2.10 (2.20–2.10)
Completeness (%)	89.6 (92.5)
Average redundancy	2.4 (2.3)
〈*I*/σ(*I*)〉	14.9 (1.6)
Rmeas (%) (Diederichs and Karplus 1997)	6.1 (68.6)
CC_1/2_ (Diederichs and Karplus 1997)	99.9 (65.5)
*R_fact_ (%)*	16.2
*R*_free._ *(%)*	23.1
Bonds (Å)	0.01
Angles (°)	2.07
Ramachandran plot	
Most favoured (%)	94.3
Allowed (%)	5.2
PDB entry code	7B1X

Values for the highest resolution shell are given in parentheses.

**Table 2:** Effect of NaCl concentration on PMGL3 esterase activity.

Concentration, M	Relative Activity * (%)
0	100
0.25	100 ± 5
0.5	86 ± 3
0.75	71 ± 2
1	67 ± 3
1.25	56 ± 1
1.5	51 ± 1
1.75	48 ± 2

* Relative activity was determined with 0.25 mM *p*-NPB at 30 °C in 50 mM Tris-HCl (pH 8.0) containing specified NaCl concentration. Data are given as means ±RSD, *n* = 3.

**Table 3:** Effect of metal ions and other additives on PMGL3 esterase activity.

Compound (1 mM)	Remaining Activity * (%)
None	100
MgCl_2_	111 ± 8
NiCl_2_	4 ± 1
CuCl_2_	3 ± 1
CoCl_2_	16 ± 4
ZnSO_4_	1.8 ± 0.1
CaCl_2_	91 ± 8
MnCl_2_	110 ± 2
EDTA	112 ± 2
NaN_3_	102 ± 2

* PMGL3 (2 µg) was incubated in 50 mM Tris-HCl (pH 8.0), 100 mM NaCl with corresponding additives at 5 °C for 30 min. Remaining activity was determined with 0.25 mM *p*-NPB at 30 °C. Data are given as means ± RSD, *n* = 3.

**Table 4:** Effect of detergents on PMGL3 esterase activity.

Detergent	Relative Activity * (%) at Concentration	
	0.05% (*w*/*v*)	0.5% (*w*/*v*)
None	100	100
Triton X-100	66 ± 1	31 ± 1
Tween 20	95 ± 6	48 ± 1
CHAPS	115 ± 2	102 ± 1
SDS	3 ± 1	3 ± 2

* PMGL3 (2 µg) was incubated in 50 mM Tris-HCl (pH 8.0), 100 mM NaCl with corresponding additives at 5 °C for 30 min. Remaining activity was determined with 0.25 mM *p*-NPB at 30 °C. Data are given as means ± RSD, *n* = 3.

**Table 5:** Effect of organic solvents on PMGL3 esterase activity.

Organic Solvent	Relative Activity * (%) at Concentration	
	5% (*w*/*v*)	10% (*w*/*v*)
None	100	100
Acetonitrile	32 ± 2	16 ± 1
Ethanol	72 ± 8	48 ± 7
Methanol	84 ± 3	54 ± 4
DMSO	97 ± 4	69 ± 1

* PMGL3 (2 µg) was incubated in 50 mM Tris-HCl (pH 8.0), 100 mM NaCl with corresponding additives at 5 °C for 30 min. Remaining activity was determined with 0.25 mM *p*-NPB at 30 °C. Data are given as means ± RSD, *n* = 3.

**Table 6:** Comparison of interfaces in T1 and T2 tetrameric forms of PMGL3. Values are estimated by PDBePISA and summarized for all interfaces within a corresponding tetramer.

	T1	T2
Total buried area, Å^2^ (% per one subunit)	6540 (27%)	4350 (18%)
Hydrogen bonds	30	16
Salt bridges	18	12
Contribution of hydrophobic interactions * (kcal/mol)	−27.6	−31.9
Free energy of assembly dissociation (kcal/mol)	13.7	6.7

* Hydrophobic interactions are estimated as solvation free energy gain upon formation of the interface.

**Table 7:** Comparison of PMGL3 structure with the structures of homologous enzymes from the GDSAG subfamily of the bHSL family.

Enzyme name	PDB ID	Q-Score	RMSD, Å	Superposed Residues_,_ % *	Sequence Identity, %	Oligomeric State **
Hormone-Sensitive Lipase EstE5 from a Metagenomic Library	3H1A	0.78	1.38	98	36	Monomer
HSL-homolog EstE7 from a metagenomic library	3K6K	0.78	1.34	98	36	Dimer
Esterase E40 from the bacterial HSL family [42]	4XVC	0.72	1.70	98	36	Monomer
HSL-like carboxylesterase from *Sulfolobus tokodaii* [38]	3AIK	0.67	1.41	93	27	Tetramer
Metagenome-derived esterase Est8 [43]	4YPV	0.64	1.61	90	25	Monomer
EstE1 carboxylesterase from a metagenomic library [40]	2C7B	0.64	1.58	92	27	Tetramer
Chloramphenicol-metabolizaing enzyme EstDL136 from a metagenomic library [44]	6AAE	0.56	1.40	82	26	Dimer
Esterase Est22 from a deep-sea metagenomic library [45]	5HC3	0.48	2.08	89	26	Dimer
Fungal esterase RmEstA from *Rhizomucor miehei* [46]	4WY5	0.59	1.69	85	23	Dimer
Fungal esterase RmEstB from *Rhizomucor miehei* [46]	4WY8	0.58	1.75	91	24	Dimer
Esterase LpEst1 from Lactobacillus plantarum [47]	4C88	0.48	1.85	86	22	16-mer/Octamer/Dimer
Esterase Est25 from a Metagenomic Library [48]	4J7A	0.49	1.95	91	23	Dimer

* A maximum percentage of matched (superposed) residues between the structures of PMGL3 and the corresponding enzyme, which lead to the lowest RMSD. ** Based on crystal contacts analysis.

**Table 8:** Characteristics of PMGL3 and the mutant variants.

Protein	*t*_1/2_, min (40 °C)	*K_m_,* mM	*k_cat_,* min^−1^	*V_max_,* mM *min^−1^ *mg^−1^	*k_cat_*/*K_m_,* min^−1^ *mM^−1^
wtPMGL3	35.7	0.98 ± 0.09	5490 ± 226	165 ± 8	5602 (100%)
D7N	51.0	0.84 ± 0.07	5103 ± 189	157 ± 8	6057 (108%)
E47Q	25.6	1.07 ± 0.09	3820 ± 124	117 ± 3	3570 (64%)
K67A	58.8	0.94 ± 0.06	3328 ± 84	102 ± 5	3523 (63%)

## Data Availability

The data presented in this study are available in the article and supplementary material.

## Supplementary Materials

The following are available online at https://www.mdpi.com/2218-273X/11/1/57/s1, S1 File. Validation report for PDB code 7B1X. S2 File. Table S1: Nucleotide sequences of the primers used for construction of the mutant genes, Table S2: Content of the secondary structure elements in wtPMGL3 and its mutant variants obtained by CD spectroscopy, Table S3. Residues stabilizing PMGL3 dimeric interface, Figure S1: Far-UV CD spectra of wtPMGL3 and its mutant variants, Figure S2: Effect of temperature on thermal stability of the wtPMGL3 and the mutants, Figure S3: A schematic plot of PMGL3 dimeric interface.
